# Mitochondrial Oxidative Phosphorylation defect in the Heart of Subjects with Coronary Artery Disease

**DOI:** 10.1038/s41598-019-43761-y

**Published:** 2019-05-20

**Authors:** Karima Ait-Aissa, Scott C. Blaszak, Gisela Beutner, Shirng-Wern Tsaih, Garrett Morgan, Janine H. Santos, Michael J. Flister, David L. Joyce, Amadou K. S. Camara, David D. Gutterman, Anthony J. Donato, George A. Porter, Andreas M. Beyer

**Affiliations:** 1 Cardiovascular Center, Department of Medicine, Med College of Wisconsin, Milwaukee, WI USA; 2 Department of Pediatrics, University of Rochester Medical Center, Rochester, NY USA; 3 Department of Physiology, Med College of Wisconsin, Milwaukee, WI USA; 4 Department of Internal Medicine, University of Utah, Salt Lake City, UT USA; 5 Genome Integrity and Structural Biology Laboratory, NIHEHS, Raleigh-Durham, NC USA; 6 Department of Surgery, Med College of Wisconsin, Milwaukee, WI USA; 7 Department of Anesthesiology, Med College of Wisconsin, Milwaukee, WI USA; 8 VA Medical Center-Salt Lake City, GRECC, Salt Lake City, Utah USA; 9 Department of Pharmacology and Physiology, University of Rochester Medical Center, Rochester, NY USA; 10 Department of Medicine (Aab Cardiovascular Research Institute, University of Rochester Medical Center, Rochester, NY USA

**Keywords:** Heart failure, Energy metabolism

## Abstract

Coronary artery disease (CAD) is a leading cause of death worldwide and frequently associated with mitochondrial dysfunction. Detailed understanding of abnormalities in mitochondrial function that occur in patients with CAD is lacking. We evaluated mitochondrial damage, energy production, and mitochondrial complex activity in human non-CAD and CAD hearts. Fresh and frozen human heart tissue was used. Cell lysate or mitochondria were isolated using standard techniques. Mitochondrial DNA (_mt_DNA), NAD + and ATP levels, and mitochondrial oxidative phosphorylation capacity were evaluated. Proteins critical to the regulation of mitochondrial metabolism and function were also evaluated in tissue lysates. PCR analysis revealed an increase in _mt_DNA lesions and the frequency of mitochondrial common deletion, both established markers for impaired mitochondrial integrity in CAD compared to non-CAD patient samples. NAD^+^ and ATP levels were significantly decreased in CAD subjects compared to Non-CAD (NAD^+^ fold change: non-CAD 1.00 ± 0.17 vs. CAD 0.32 ± 0.12* and ATP fold change: non-CAD 1.00 ± 0.294 vs. CAD 0.01 ± 0.001*; N = 15, P < 0.005). We observed decreased respiration control index in CAD tissue and decreased activity of complexes I, II, and III. Expression of ETC complex subunits and respirasome formation were increased; however, elevations in the de-active form of complex I were observed in CAD. We observed a corresponding increase in glycolytic flux, indicated by a rise in pyruvate kinase and lactate dehydrogenase activity, indicating a compensatory increase in glycolysis for cellular energetics. Together, these results indicate a shift in mitochondrial metabolism from oxidative phosphorylation to glycolysis in human hearts subjects with CAD.

## Introduction

Mitochondria are the major source of cellular energy in the heart, producing ATP via the electron transport chain (ETC). Mitochondrial damage can impair cellular function and has been linked to several cardiovascular diseases^1–4^. For example, in coronary artery disease (CAD)^5^, studies have identified an increased frequency of the mitochondrial DNA (_mt_DNA) common deletion (typically, a well-characterized 4,977 base pair deletion) and elevated _mt_DNA damage when compared to healthy controls^6,7^. Similarly, a relative rise in _mt_DNA deletion frequency was found in human heart tissue with chronic ischemia^1^. Although CAD is associated with _mt_DNA damage and deletions, the specific effect on mitochondrial function has not been examined.

The mammalian _mt_DNA is a small genome of 16,000 base pairs that encodes for proteins of the ETC (13 subunits of complexes I, III,IV, and the ATP synthase (complex V)) that are essential for oxidative phosphorylation (OXPHOS), and various non-coding RNA and signaling peptides critical for normal mitochondrial function^8^. During mitochondrial respiration, electrons from complexes I and III can react with molecular oxygen to form superoxide, a damaging reactive oxygen species (ROS)^9^. Because of its lack of histones, which protect against oxidative stress and its close proximity to the ETC, the primary source of ROS, _mt_DNA is more vulnerable to the accumulation of ROS-induced damage than nuclear DNA^10–13^.

Oxygen-consuming mitochondrial OXPHOS is the physiological means of ATP production in the heart. Several animal models and human studies have demonstrated that energy demands in the failing heart are increasingly dependent on glycolysis^14^. However, a detailed characterization of the cellular changes occurring in the cardiac tissue of human CAD patients is lacking.

Previous studies from our group have shown an increased level of _mt_ROS in CAD patients as determined by the increase release of mitochondrial H_2_O_2_, a very important pro-inflammatory signaling factor^15,16^. Thus, we hypothesized that as a consequence of this rise of _mt_ROS in human tissue from patients with CAD, we would observe increased _mt_DNA damage, reduced OXPHOS, decreased mitochondrial respiration, and a corresponding rise in glycolytic flux. The goal of this study was to determine the bioenergetic profile of cardiac mitochondria in tissue from subjects suffering from CAD compared to non-CAD controls.

## Materials and Methods

### Tissue acquisition and general protocol

The study was performed in accordance with the ethical principles for medical research involving human subjects. The Institutional Review Board of the Medical College of Wisconsin and Froedtert Hospital deemed collected or otherwise discarded tissue to be exempt and as a result no patient consent was required. The samples were collected from surgical discarded tissues and rejected donor hearts in areas without any obvious sign of infarct or other damage. Whole human hearts were obtained in de-identified fashion and placed in cold 4 °C HEPES (NaCl 275 mM, KCL 7.99 mM, MgSO_4_ 4.9 mM, CaCl_2_·2H_2_O 3.2 mM, KH_2_PO_4_ 2.35 mM, EDTA 0.07 mM, glucose 12 mM HEPES acid 20 mM) buffer solution. Atrial and ventricular tissues from subjects without known cardiovascular risk factors or clinical diagnosis of CAD were used for Non-CAD groups.

### Colorimetric determination of NAD + levels

The NAD + levels were measured in heart lysates from CAD and non-CAD patients. The experiments were performed using NAD/NADH-Glo^TM^ Assay (Promega Corporation, Madison WI, USA) according to the manufacturer’s protocols.

### Fluorometric determination of ATP levels

The ATP levels were measured in heart lysates from CAD and non-CAD patients. The experiments were performed using Luminescence Assay kit (Biovision Incorporated, Milpitas CA, USA) according to the manufacturer’s protocols.

### Mitochondrial isolation

Sections of fresh or frozen CAD and non-CAD hearts were used to isolate intact mitochondria or mitochondrial proteins, respectively. The tissues were rapidly immersed in 4 °C cold isolation buffer [200 mM mannitol, 50 mM sucrose, 5 mM KH_2_PO_4_, 5 mM 3-(N-morpholino) propanesulfonic acid, and 1 mM EGTA, with 0.1% bovine serum albumin, pH 7.15] and then minced into 1 mm^3^ pieces. The tissue suspension was initially homogenized for 15 s in 2.5 ml of isolation buffer containing 5 U/ml protease (P5459, SIGMA Life science) and for another 15 s after addition of 17 ml of isolation buffer. Mitochondria were then isolated by differential centrifugation at 4 °C, as described previously^17^. First, the suspension was centrifuged at 8,000 *g* for 10 min; the resulting pellet was then re-suspended in 25 ml of isolation buffer and centrifuged again at 750 *g* for 10 min to remove cellular debris. Next, the supernatant containing the mitochondrial fraction was further centrifuged at 8,000 *g* for 10 min, and the final pellet was suspended in 0.5 ml of isolation buffer and kept on ice for experimentation. Protein concentration was determined by the Bradford method with BSA as a standard.

### Mitochondrial respiration

Mitochondria isolated from fresh cardiac tissue as described above were used to measure mitochondrial respiration using Clark-type O_2_ electrode (model 1302; Strathkelvin Instruments, Glasgow, Scotland) in a chamber (model MT200A, Strathkelvin Instruments) as previously described^17^. Briefly, mitochondria were suspended in experimental buffer and loaded to the chamber of the oxygraph in the presence of potassium-pyruvate/malate (KPM; 10 mM final concentration) or sodium (Na) based succinate (SUC, 10 mM final concentration) and oxygen consumption monitored (state 2 respiration). Adenosine diphosphate (ADP, 250 μM final concentration) was then added to induce the higher rate of oxygen consumption (state 3 respiration), which is followed by a lower rate of oxygen consumption after the added ADP was depleted (state 4 respiration). The intactness of OXPHOS or coupling was appraised by determining the respiratory control index (RCI), the ratio of the maximum oxygen consumption rate, state 3 respiration, to the oxygen consumption rate after most of the added ADP is converted to ATP, state 4 respiration. The purity of mitochondria was not assessed in this experiment as only intact mitochondria are able to respire.

### In-gel assay of mitochondrial ETC complexes I-V activities

The activities of the mitochondrial electron transport chain complexes were measured using a native gel-based assay as previously described^18^. Briefly, mitochondrial proteins isolated as described above from frozen cardiac tissue were solubilized by addition of 10% dodecyl maltoside and 10% digitonin. After centrifugation at 16,000 *g*, the supernatant was loaded on 2 separate native gels. At the end of the run, one gel was used as a loading control for total protein and stained with Coomassie blue and the second gel was stained for activity. Each complex was stained with the appropriate substrate to reveal its activity, as shown in Supp Table 4. Image J software was used to quantify the density of the activity-bands and normalize them to the corresponding total protein loaded on the Coomassie gel.

### Mitochondrial OXPHOS and supercomplex assembly and activity analysis

#### Mitochondrial isolation

Sections of frozen CAD and Non-CAD hearts (~0.15 g) were thawed on ice and then immediately immersed and rinsed in 4 °C cold isolation buffer 225 mM mannitol, 75 mM sucrose, 0.5 mM EGTA, 0.5 mM EDTA, 10 mM Tris, pH 7.4. The tissue was minced and homogenized with an Elvehjem potter. The homogenized tissue was centrifuged for 5 minutes at 1000 *g* to remove tissue fragments and debris. The supernatant was transferred into fresh tubes and the sediment was homogenized and centrifuged again for 5 minutes at 1000 *g*. The supernatant was transferred into fresh tubes, and then centrifuged for 10 minutes at 12,000 *g*. The mitochondria-enriched sediment was re-suspended in 100–150 µl of isolation buffer. Total protein concentration was determined using a BCA kit from Pierce with BSA as a standard. All isolation procedures were carried out at 4 °C.

### SDS and clear native electrophoresis

Denaturing and Clear Native (CN) electrophoresis was done according to published protocol^19,20^. Denatured proteins were separated on 16% SDS gels. For CN electrophoresis, aliquots of 20 µg protein were re-suspended in extraction buffer (50 mM NaCl, 50 mM Imidazole (pH 7 at 4 °C), 2 mM aminocaproic acid and 1 mM EDTA). Mitochondrial proteins were extracted with 3 µg n-dodecyl-beta-D-maltopyranoside/µg protein for 15 minutes on ice. After centrifugation at 14,000 g to remove tissue fragments, protein complexes were loaded onto CN gradient gels (3–10%) and separated at 200 V for 115 minutes at 4 °C. The separated proteins/protein complexes were transferred onto nitrocellulose membranes, incubated with the primary antibody and a horseradish peroxidase coupled secondary antibody. Labeling was detected by enhanced chemi-luminescence or Supersignal West Dura (Pierce, Rockport Il).

### Antibodies used

ATP5A (clone 15H4C4, 1:2000, 43–9800 Thermofisher), NDUFB6 (clone 21C11BC11, 1:1000, ab110244 Abcam), VDAC (1:2000, clone 31HL, 529534 Calbiochem).

### Enzymatic activity determination of complex I

The activity of Complex I was measured with a Spectramax 384 plus microplate reader (Molecular Devices, Sunnyvale CA) at room temperature using published protocols^21,22^. The enzymatic activity was normalized to mg protein and expressed as milli units/mg (mU/mg). Generally, the test-volume was 0.2 ml, and 20–50 µg of protein was used. Samples were frozen and thawed twice prior to testing. Complex 1 activity was measured as NADH-ubiquinone oxidoreductase (340 nm; ε: NADH 6.81 mM^−1^cm^−1^), or NADH-cytochrome *c* oxidoreductase (550 nm, ε for cytochrome *c*: 18.7 mM^−1^cm^−1^) activity. Where necessary, the test was repeated with 2 µg/ml rotenone to assess mitochondria specific, rotenone-sensitive activity. The active and de-active form of Complex I was accessed by incubating a mitochondrial aliquot with and without 1 mM N-ethyl-maleimide (NEM) in 0.25 M sucrose, 20 µM NADH, 50 mM Tris-HCl, 0.2 mM EDTA pH 7.0 for 10 minutes^23,24^. The Complex I activity was then measured as NADH-ubiquinone oxidoreductase assay and 200 µM NADH was used to start the reaction.

### Pyruvate kinase activity assay

Pyruvate kinase activity was measured in heart lysates from CAD and non-CAD patients. The experiments were performed using the Pyruvate Kinase Assay Kit (ab83432, Abcam, Cambridge, MA, USA) according to the manufacturer instructions.

### Lactate dehydrogenase activity assay

Lactate Dehydrogenase activity (LDH) was measured in heart lysates from CAD and non-CAD patients. The experiments were performed using the LDH Kit (ab102526, Abcam, Cambridge, MA, USA) according to the manufacturer instructions.

### Western blot analysis

Protein expression was analyzed as previously described^25^. Briefly, frozen human left ventricles (LV) in each group were homogenized in an ice-cold lysis buffer containing protease and phosphatase inhibitors (5872 S; Cell Signaling), centrifuged and the supernatant was transferred to a new tube. Total protein concentration was determined using a BCA protein assay kit according to the manufacturer’s instructions (23225; Thermo Scientific). Lysates were loaded and separated using SDS-PAGE and then transferred onto a PVDF membrane. The following antibodies were used in the present study: anti- phosphorylated (S616) (1:1000; 3455; Cell Signaling) and total Dynamin-like protein 1 (DRP1) (1:1000; ab56788; Abcam) anti- Mitofusin 1 (MFN1) (1:2000; ab57602; Abcam), anti-Mitofusin 2 (MFN2) (1:2000; ab56889; Abcam), anti- Optic Atrophy 1 (OPA1) (1:1000; 612606; DB Biosciences), total OXPHOS human WB antibody cocktail (1:2000; ab110411; Abcam), anti-ATP5A subunit (1:1000; sc-136178; Santa Cruz) and anti-GAPDH (1:1000; ab8245; Abcam).

### Mitochondrial DNA damage analysis

Quantitative PCR (qPCR) was used to assay _mt_DNA damage as described previously^26^. Briefly, total mitochondrial DNA was extracted using QIAGEN Genomic Tip and Genomic DNA Buffer Set Kit (QIAGEN, Valencia, CA). Purified genomic mitochondrial DNA was quantified fluorometrically using Pico Green dsDNA reagent (Molecular Probes, Life Technologies, USA). Lambda (λ)/ HindDIII DNA (Gibco Invitrogen, Paisley, UK) was used to generate a standard curve and adjust the final DNA concentration to 3 ng/μL. The “hot start” PCR used the Gene Amp XL PCR Kit (Applied Biosystems, Foster City, CA, USA) with 15 ng DNA, 1X buffer, 100 ng/μL BSA, 200 μM dNTPs, 20 pmol of each primer (Small fragment: Sense 5′-CCC CAC AAA CCC CAT TAC TAA ACC CA-3′, Antisense: 5′-TTT CAT CAT GCG GAG ATG TTG GAT GG-3′; Large fragment: Sense: 5′-TCT AAG CCT CCT TAT TCG AGC CGA-3′, Antisense: 5′-TTT CAT CAT GCG GAG ATG TTG GAT GG-3′), 1.3 mM Mg^++^ and H_2_O. The reaction was brought to 75 °C before adding 1U/reaction enzyme. Specific primers were used to amplify a large fragment of _mt_DNA (8.9 kb) to determine _mt_DNA integrity; and a small fragment (139 bp) of the mitochondrial genome to monitor changes in _mt_DNA copy number and to normalize the data obtained when amplifying the 8.9-kb fragment. Relative amplifications were calculated to compare CAD hearts to non-CAD hearts. These values were then used to estimate mathematically, assuming a Poisson distribution as previously described^26^, the number of lesions present in _mt_DNA.

### Mitochondrial DNA common deletion

LV tissues from CAD and Non-CAD subjects were used to extract genomic _mt_DNA as explained above. To screen for common deletion in _mt_DNA, qPCR analysis was performed using two pairs of primers as follows: Sequence-independent qPCR with RT² SYBR Green qPCR Mastermix (Qiagen, Inc.) was performed according to the manufacturer’s protocol to determine relatively quantitative standard curve derived signals for “low deletion” and “high deletion” regions of human mitochondrial genome in all samples. The targeted “high deletion” region is the region of mitochondrial genome that includes site of “common deletion.” Low deletion region F-CCCGGTAATCGCATAAAACTTAAAACTT and R-TAAGAAGAGGAATTGAACCTCTGA CTGTAA; and high deletion region F-CCCTAACTCTGGCCTATGAGTGAC and R-ACGAATTCGGTTCAGTCTAATCCTTTTTG.

Relative quantitative standard curves were generated by dilution series of standard pool human DNA. Low deletion region signal was used to control for _mt_DNA content in samples, and common deletion load per genome was expressed as the ratio of high deletion to low deletion content. All samples were assayed in triplicate, and replicate means were used for analysis.

### RNA-sequencing

Total RNA was collected from left ventricles of non-CAD (control) subjects (n = 7) and CAD patients (n = 8) using TRIzol Reagent (ThermoFisher, 15596-026). Total RNA (4 µg) was poly-A purified, reverse-transcribed, and chemically fragmented using Illumina’s TruSeq RNA library kit, per the manufacturer’s protocol. Individual libraries were prepared for each sample, indexed for multiplexing, and then sequenced on an Illumina HiSeq. 2500 (Illumina, Inc., San Diego, CA). Reads of each sample were aligned to NCBI Build GRCh38.p2 of the human transcriptome references using Bowtie2 version 2.2.3^27^. Default parameters were used with the exception of a Bowtie2 offset of 1, trading index size for increased alignment speed. Sequences for all RNA transcripts were annotated using NCBI Homo Sapiens Annotation Release 107. Expression abundances were quantified at the whole transcript-level as effect counts using eXpress version 1.5.1^28^. The transcript-level count data were aggregated per gene and rounded to an integer to produce gene-level count matrix. Differential expression (DE) analysis was performed with the Bioconductor package DESeq. 2 version 1.12.4^29^ to compute log 2 fold changes and FDR-adjusted p-values. Statistical significance was determined at an FDR threshold of 0.05. Data were analyzed for molecular and functional pathway enrichment using the Ingenuity IPA tool (Qiagen, Redwod City, CA) and the STRING protein-protein interaction network database (https://string-db.org/).

### Statistical methods

Data are presented as mean ± SEM. Differences between groups at each experiment were determined using a Student’s t-test. A probability value of p < 0.05 was considered to be statistically significant. Statistical analyses were performed using Graphpad Prism version 7 software.

## Results

### Study population and sample acquisition

A total of 57 heart samples were used for this study: 22 of the samples were obtained from Non-CAD and 35 from patients with CAD. The full clinical characteristics of both groups are detailed in Supp Table 1.

As viability of mitochondria is mandatory for the mitochondrial respiration studies, most samples used for direct measurement of mitochondrial oxygen consumption were collected from otherwise atrial discarded appendages within 1–2 hours after removal of the tissue during cardiopulmonary bypass surgery. Other experiments were performed in previously frozen cardiac tissue.

### Decreased mitochondrial DNA integrity associated with CAD

**Figure 1:**
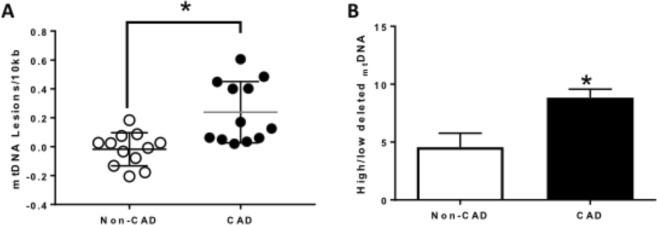
_mt_DNA integrity analysis in human hearts. (**A**) _mt_DNA lesions level measured in _mt_DNA isolated from left ventricle of human hearts; n = 12 in each group; *p < 0.05 for hearts from CAD subjects vs. hearts from Non-CAD subjects. (**B**) _mt_DNA deletion level quantified in _mt_DNA isolated from left ventricle of human hearts from CAD patients compared to Non-CAD subjects; Values are expressed as mean ± SEM expressed as arbitrary units; n = 8 in each group; *P < 0.05 t student test.

To assess _mt_DNA integrity, total genomic mitochondrial DNA was isolated from LV of subjects with and without CAD and used to perform PCR-based assays of _mt_DNA damage and the mitochondrial common deletion. Mitochondrial DNA lesions increased significantly in LV from CAD patients compared to non-CAD (N = 12; p = 0.001) (Fig. 1A). Furthermore, when similar groups of samples were analyzed for a common _mt_DNA deletion, CAD LV samples displayed a higher frequency of the _mt_DNA common deletion compared to non-CAD LV samples (N = 8; p = 0.007) (Fig. 1B).

### Modulation of mitochondrial dynamics-related proteins in CAD subjects

**Figure 2:**
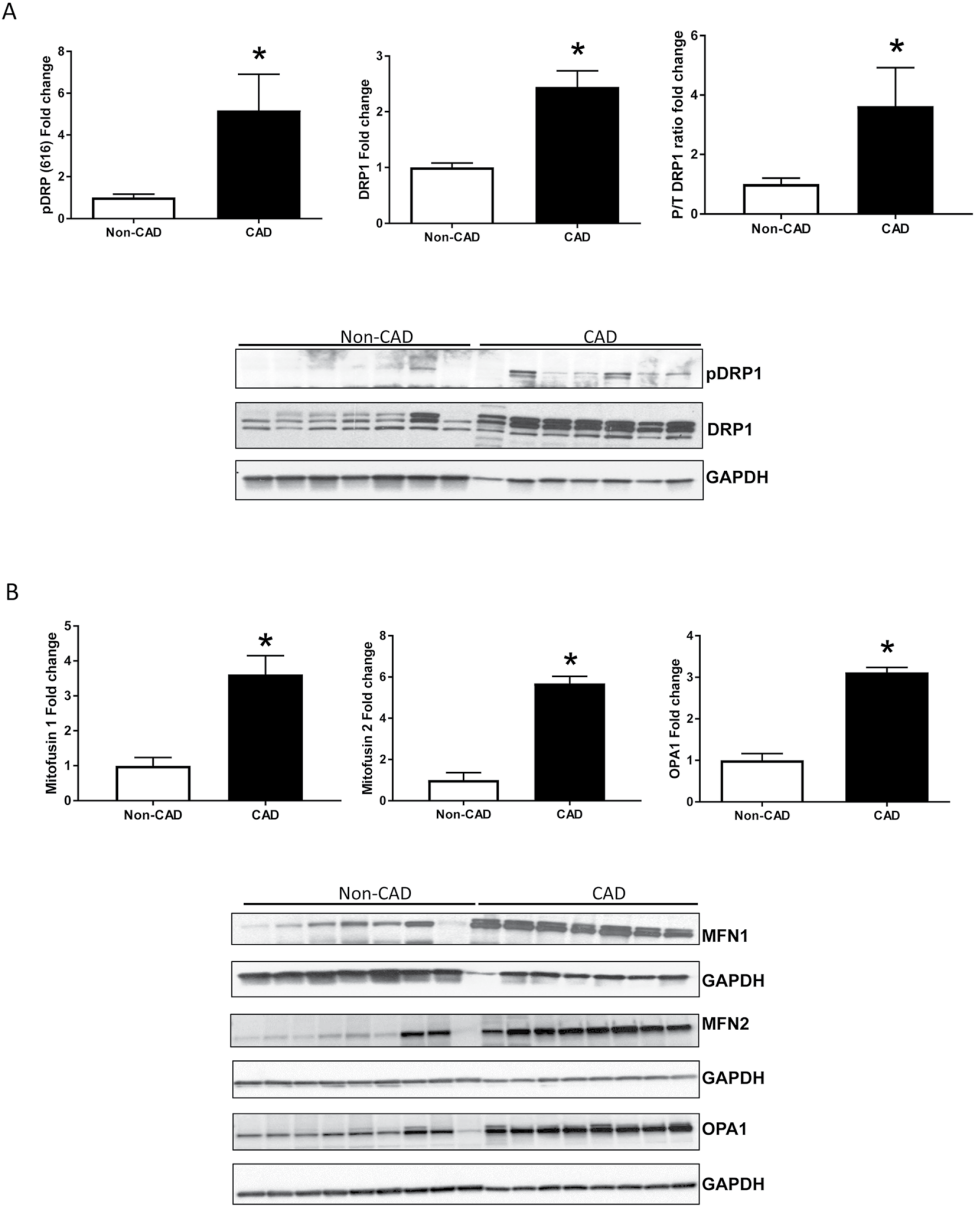
Mitochondrial dynamic markers expression in left ventricles of human hearts. (**A**) Mitochondrial fission markers expression: phosphorylated DRP (left panel), total DRP (middle panel) and phosphorylated to total ratio DRP (right panel); expression analyzed by western blot in human hearts from CAD compared to Non-CAD; n = 7 in each group; *P < 0.05 t student test vs. Non-CAD human hearts. (**B**) Mitochondrial fusion markers expression: Mitofusin 1 (left panel), Mitofusin 2 (middle panel) and OPA1 (right panel); expression analyzed by western blot in human hearts from CAD compared to Non-CAD; Values are expressed as mean ± SEM expressed as fold change relative to the Non-CAD; n = 8 in each group; *P < 0.05 t student test vs. Non-CAD human hearts.

Mitochondria are dynamic organelles that change their morphology by fusion and fission, especially following stress^30,31^. Altered fusion and fission of mitochondria are associated with elevated levels of ROS production and have been linked to the development of various cardiovascular phenotypes in animal models^32^. To evaluate the mitochondrial dynamics associated with CAD, we measured expression of markers regulating mitochondrial dynamics: Drp1, Mfn1, Mfn2 and Opa1 (Fig. 2). Both fission (phosphorylated and total DRP1) (Fig. 2A) and fusion (MFN1, MFN2, OPA1) (Fig. 2B) markers increased significantly in the LV hearts from CAD subjects compared to non-CAD controls (P/T DRP1: Non-CAD 1 ± 0.2 and CAD 3.6 ± 1.3* n = 7; MFN1: Non-CAD 1 ± 0.2 and CAD 3.6 ± 0.5*; MFN2: Non-CAD 1 ± 0.4 and CAD 5.6 ± 0.3, n = 9; *p < 0.05 for CAD vs Non-CAD LV lysates). These data suggest consistent changes in mitochondrial dynamics in the CAD hearts.

### Reduced mitochondrial energy production in CAD

**Figure 3:**
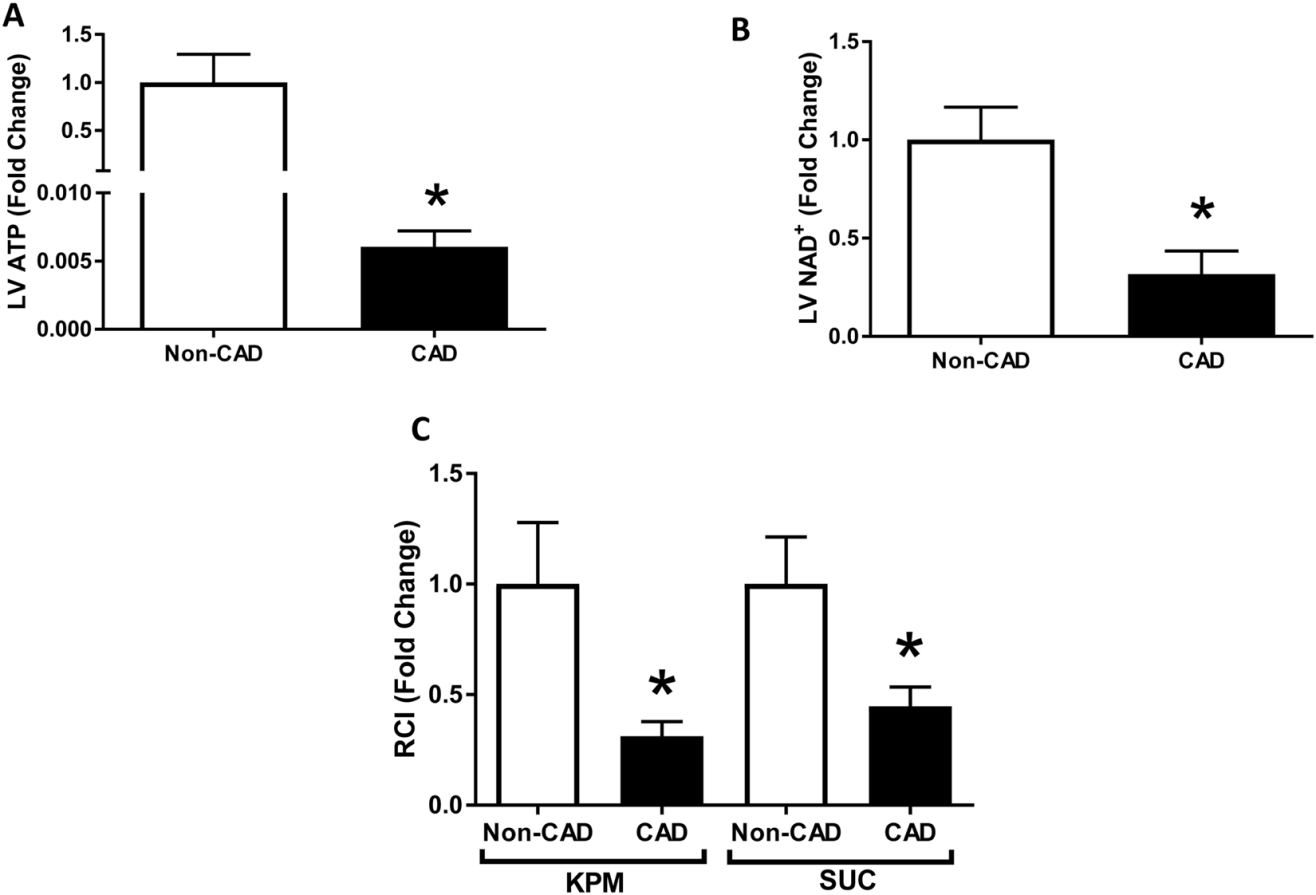
Mitochondrial bioenergetics in human hearts. (**A**,**B**) ATP and NAD^+^ levels were measured in left ventricles of human hearts; n = 15 in each group; *p < 0.05 vs Non-CAD hearts. (**C**) Respiratory Control Index (RCI) of isolated mitochondria of fresh human hearts in the presence of potassium pyruvate-malate (KPM) or Succinate (SUC); n = 5 in each group; *p < 0.05 vs Non-CAD hearts. Values are expressed as fold change mean ± SEM relative to Non-CAD mitochondrial respiration.

Mitochondria are the main source of ATP in cardiac cells, and oxidative stress is known to impair ATP generation^33,34^. We measured intracellular ATP as an indicator of mitochondrial function in the LV tissue lysates from both CAD and non-CAD subjects. A drastic decrease in ATP levels in the LV lysates from CAD subjects compared to non-CAD subjects (Fig. 3A) was observed. Similar patterns were observed in the right ventricle and in the left and right atria and of the heart from these same subjects (Supp Fig. 1A–C).

As NAD + plays a central role in mitochondrial respiration by acting as a coenzyme for oxidoreductases and dehydrogenases^35–37^, we measured its levels in LV tissue lysates from both groups. NAD + levels were significantly decreased in CAD LV samples (Fig. 3B). Similar changes were observed in left and right atria as well as in the right ventricle of the heart (Supp Fig. 1D–F).

To directly assess mitochondrial respiratory capacity and the effectiveness of OXPHOS to convert ADP to ATP, we measured oxygen consumption rate in mitochondria isolated from fresh cardiac tissue of CAD and non-CAD subjects using an oxygraph. Figure 3C shows the results of the RCIs obtained using complex I substrate, KPM, and complex II substrate, SUC. The RCIs were significantly lower in the mitochondrial samples from CAD hearts compared to non-CAD control hearts (KPM-RCI: Non-CAD 11.5 ± 3.2 and CAD 3.6 ± 0.8; SUC-RCI: Non-CAD 10.8 ± 2.3 and CAD 4.8 ± 0.9, n = 5; *p < 0.05). These data suggest that the deficit in ATP observed in the hearts from CAD subjects is caused, at least in part, by a significant defect in the mitochondrial OXPHOS machinery, suggesting that the observed reduction in ATP levels arises from impaired mitochondrial respiration.

**Figure 4:**
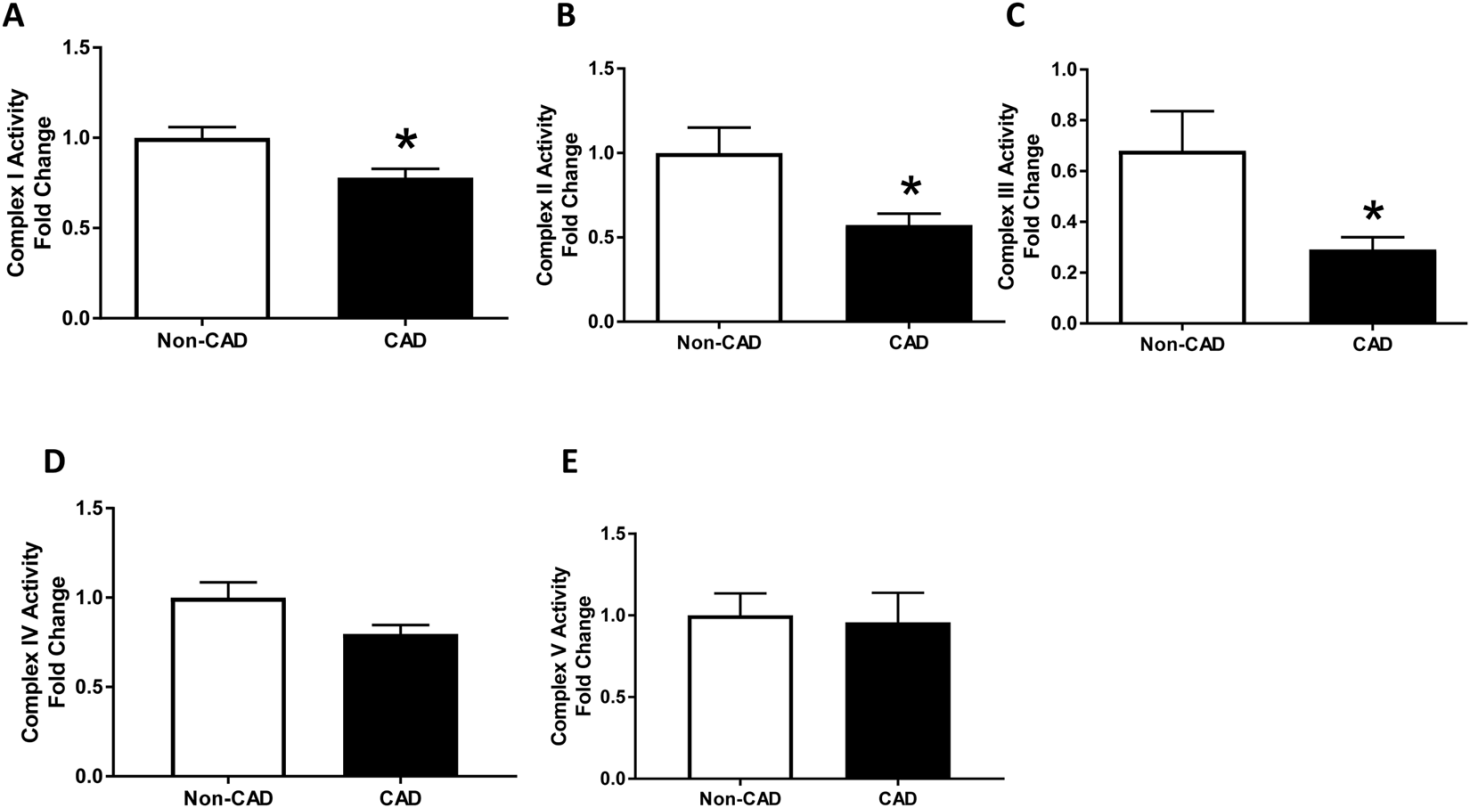
Mitochondrial Electron Transport Chain Complexes Activity. (**A**) Mitochondrial Complex I (n = 7 in each group); (**B**) Complex II (n = 7 in each group); (**C**) Complex III (n = 7 in each group); (**D**) Complex IV (n = 7 in each group); (**E**) Complex V (n = 7 in each group); all measured in isolated mitochondrial lysates of fresh human hearts; Values are expressed as mean ± SEM expressed as fold change relative to the Non-CAD; *p < 0.05 t student test vs Non-CAD heart mitochondrial extracts.

To further study the effect of CAD on mitochondrial respiration, we evaluated the activity of each complex (I-V) of the ETC in in-gel assays of native gel. Consistent with the mitochondrial respiration results, complexes I-III respective activities were markedly decreased in mitochondria isolated from LV tissues of patients with CAD (Fig. 4A–C). (Complex I: Non-CAD 1 ± 0.1 and CAD 0.7 ± 0.05, n = 7; Complex II: Non-CAD 1 ± 0.2 and CAD 0.6 ± 0.1, n = 9; Complex III: Non-CAD 1 ± 0.3 and CAD 0.4 ± 0.1, n = 7) in relation to non-CAD subjects, suggesting a significant dysfunction within the ETC in CAD. There were no significant differences in complex IV and V activity between the two groups (Fig. 4D,E).

**Figure 5:**
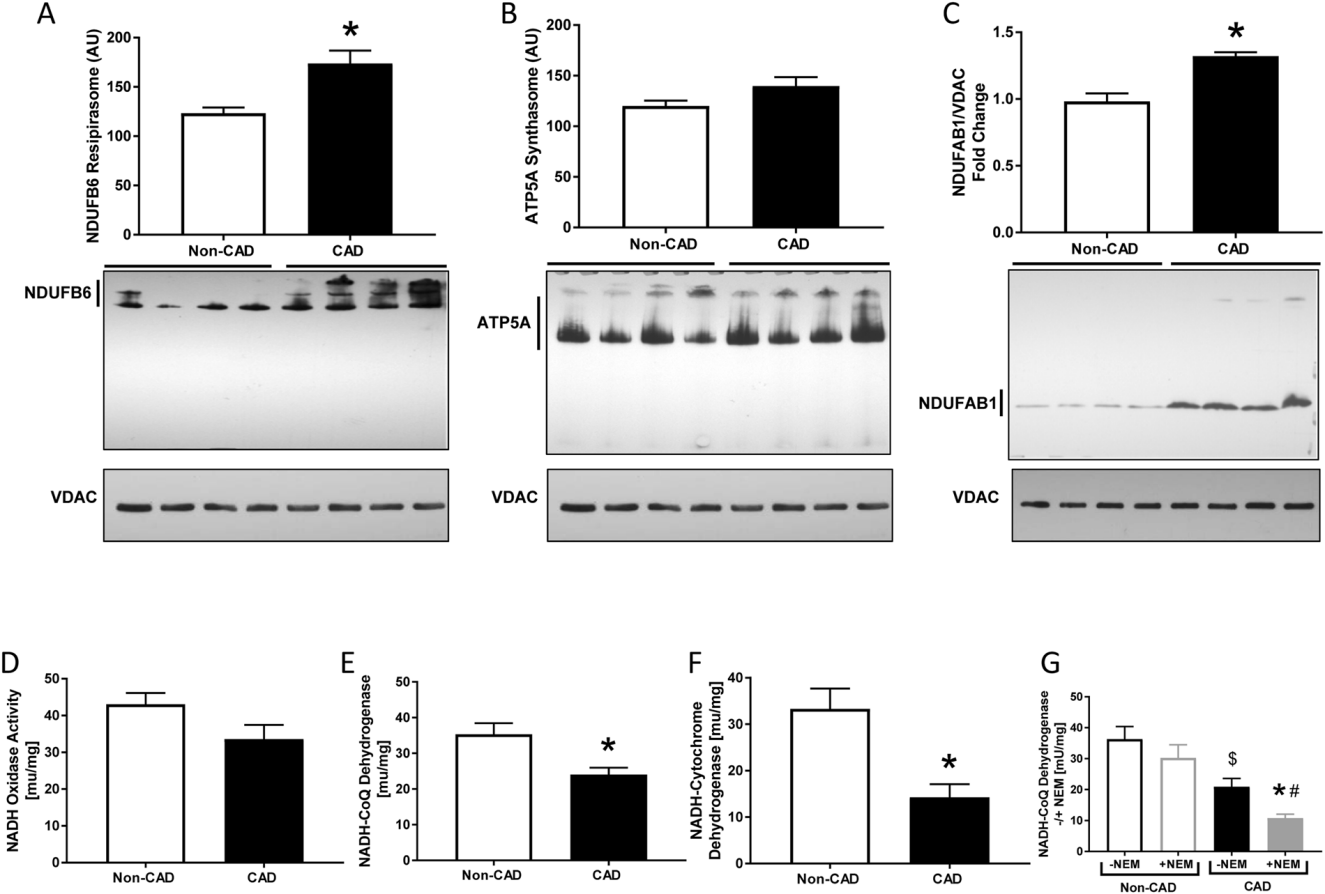
Supercomplexes activity and formation in human hearts. (**A**) Supercomplex I (Respirasome) formation (n = 4 in each group) (**B**) Supercomplex II (Synthatosome) formation (n = 4 in each group), (**C**) Complex I NDUFAB1 subunit expression (n = 4 in each group), (**D**) NADH Oxidase Activity (n = 8 in each group), (**E**) NADH-ubiquinone dehydrogenase activity (n = 8 in each group), (**F**) NADH-cytochrome c oxidoreductase, (**G**) NADH-ubiquinone dehydrogenase activity in the presence and absence of N-ethylmaleimide (NEM), (n = 8 in each group), Values are expressed as mean ± SEM in arbitrary unit (AU); *p < 0.05 t student test vs Non-CAD heart extracts.

To determine if CAD alters ETC assembly, we examined the presence of mitochondrial supercomplexes, which consist of either complexes I, III and IV (respirasomes) or ATP synthase complex (synthasomes). A decline in respirasomes has been observed due to aging or IR injury^38^, while synthasomes assemble due to bioenergetic demands^39^. We separated mitochondrial protein complexes by CN electrophoresis and analyzed supercomplex levels by immunoblotting^19^. We did not find changes in the presence of oligomeric ATP synthase in CAD patients relative to Non-CAD patients (Fig. 5B). However, using an antibody against the complex I subunit NDUFB6, we found more respirasomes in an enriched mitochondrial fraction obtained from frozen heart tissue of CAD patients relative to Non-CAD patients (Fig. 5A, n = 4, p ≤ 0.013). In addition, the levels of the complex I subunit NDUFAB1 were higher in denaturing immunoblots of CAD patients (Fig. 5C, n = 8, p ≤ 0.004) suggesting higher levels of complex I and its assembly into respirasomes.

Since we had found decreased complex I activity but increased complex I expression and assembly, we then tested complex I activity and the connectivity of complex I to ubiquinone and cytochrome c using spectrophotometry. First, we measured the activity of complex I to oxidize electrons and to transfer them to ubiquinone (Fig. 5D–G). While the NADH oxidase activity of complex I only trended to a decrease (Fig. 5D, n = 8, p = 0.069), its activity as NADH-ubiquinone dehydrogenase was markedly decreased in CAD patients (Fig. 5E, n = 8, p ≤ 0.008). Second, the transfer of electrons from complex I to complex III, when measured as NADH-cytochrome c oxidoreductase, was significantly decreased in CAD patients (Fig. 5F, n = 8, p ≤ 0.003). Third, we used N-ethylmaleimide (NEM), which can bind only to de-active complex I to prevent its activation by NADH^40,41^. We found that even though more complex I was expressed and assembled, this ETC complex was more likely to be in its de-active state in CAD samples (Fig. 5G; n = 8, p ≤ 0.004) indicating a higher content of de-active form of complex I.

### Change in regulators of cellular and mitochondrial metabolism in CAD

**Figure 6:**
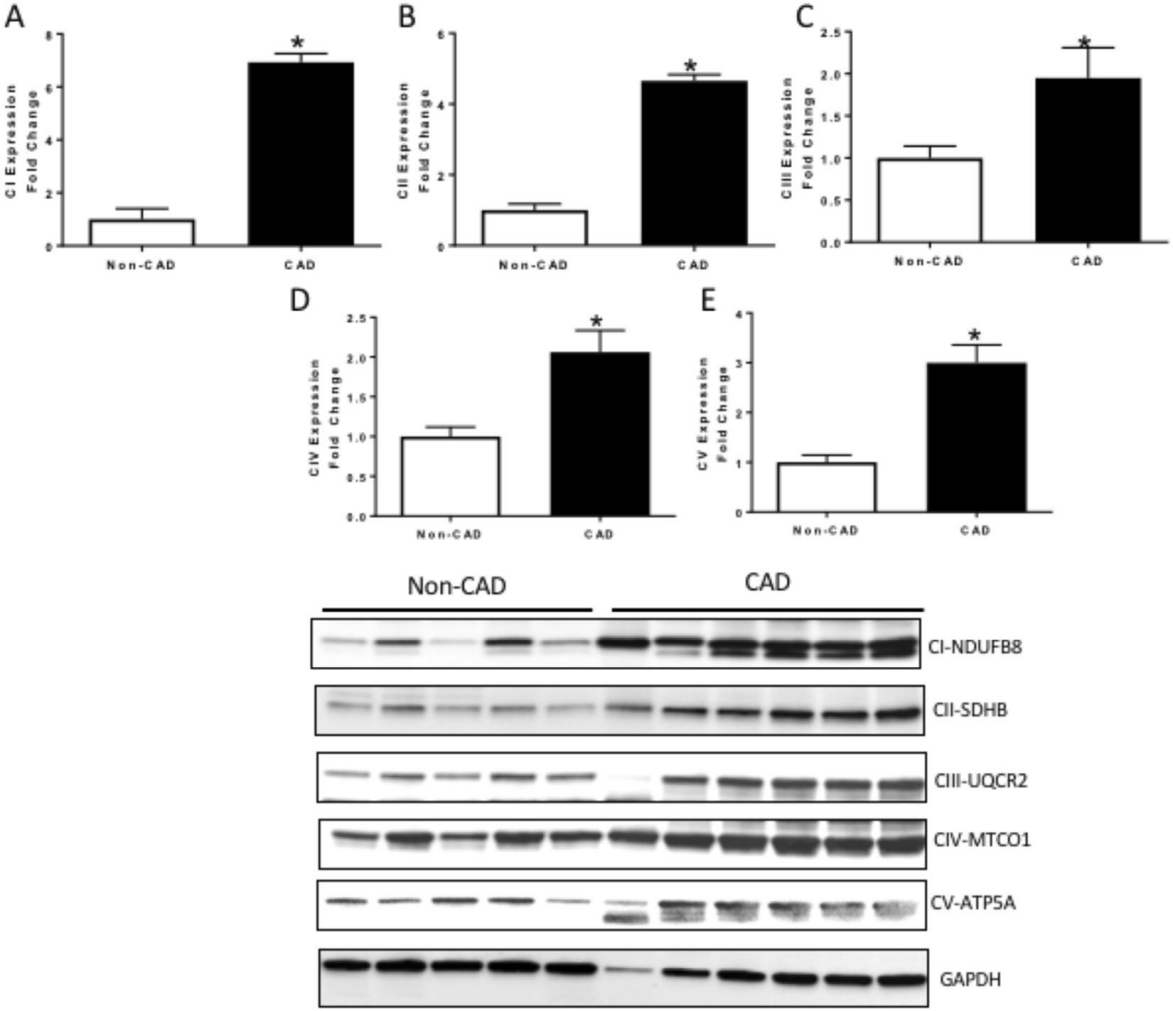
Oxidative phosphorylation complexes expression in human hearts. (**A**) Nuclear coded NDUFB8 Complex I-subunit expression; (**B**) Nuclear coded SDHB Complex II-subunit; (**C**) Nuclear coded UQCR2 Complex III-subunit; (**D**) Mitochondrial coded MTCO1 Complex IV-subunit; (**E**) Nuclear coded ATP5A Complex V-subunit; all measured by western blot from whole lysates of human hearts; Representative blots are originated from different exposure times of the same blot using an antibody cocktail. Values are expressed as mean ± SEM expressed as fold change relative to the Non-CAD; n = 8 in each group; *p < 0.05 t student test vs Non-CAD heart extracts.

We then further tested whether the _mt_DNA damage, decline in ATP levels, and reduced complex activity observed in CAD patients was associated with impaired production of key subunits of the ETC, where the major energy generation occurs through OXPHOS. As shown in Fig. 6, protein levels of NADH: Ubiquinone Oxidoreductase Subunit B8 (NDUFB8; complex I) (Fig. 6A), Succinate dehydrogenase [ubiquinone] iron-sulfur subunit (SDHB; complex II) (Fig. 6B), Ubiquinol-Cytochrome C Reductase Core Protein 2 (UQCRC2; complex III) (Fig. 6C), complex IV mitochondrial cytochrome c oxidase I (MT-CO1) (Fig. 6D) and mitochondrial ATP synthase 5 A (ATP5A; complex V) (Fig. 6E); all showed increased expressions in the CAD compared to the non-CAD LV lysates (N = 6, P < 0.05). Although the antibody used consists of a cocktail of antibodies for the 5 different subunits, it was difficult to represent the blot as a whole with one exposure time (see Supp Fig. 3). Thus, we chose to show every row corresponding to each subunit individually and, as results for ATP5A were inconsistent using the cocktail of antibodies (see Supp Fig. 2), we probed different immunoblots with antibodies to this protein separately for this analysis

### Compensatory increase in glycolysis in subjects with CAD

**Figure 7:**
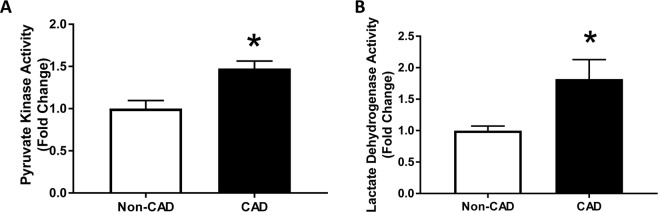
Glycolytic pathway in human hearts. (**A**) Pyruvate Kinase Activity and (**B**) Lactate Dehydrogenase Activity were measured in lysates of left ventricles of human hearts; Values are expressed as mean ± SEM expressed as fold change relative to Non-CAD heart lysates; n = 8 in each group; *p < 0.05 vs Non-CAD hearts.

As OXPHOS activity was decreased, we hypothesized that the heart of patients with CAD increasingly relies on glycolysis to produce ATP. To test this hypothesis, we assessed the pyruvate kinase activity, the final step of glycolysis that yields an additional ATP. In LV lysates from CAD subjects, pyruvate kinase activity was increased significantly compared to Non-CAD LV lysates (Fig. 7A) (Non-CAD 1 ± 0.1 and CAD 1.5 ± 0.09, n = 8, p < 0.05). These results suggest a switch in the metabolic profile from mitochondrial OXPHOS to glycolysis in CAD hearts.

In a parallel experiment, we tested LDH activity, a key enzyme that reduces pyruvate to lactate and regenerates cytosolic NAD + from NADH. Like the pyruvate kinase activity (Fig. 7B), the samples from LV of CAD subjects displayed an increased activity of LDH compared to the Non-CAD subjects (Non-CAD 1 ± 0.1 and CAD 1.8 ± 0.3, n = 12, p < 0.05). The results further point to metabolic switch towards more reliance on glycolysis as the major source of ATP with concomitant increase in lactate production in the CAD hearts.

### Molecular pathways are altered by CAD

**Figure 8:**
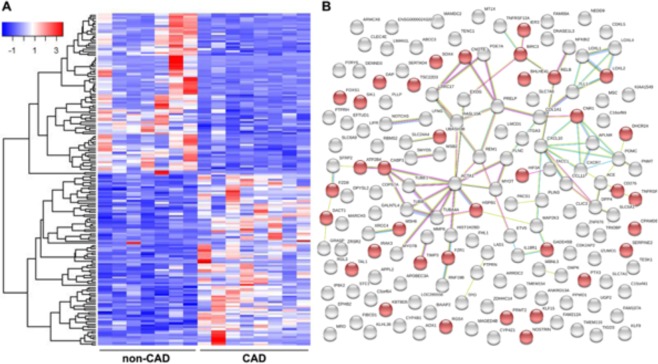
Gene network analysis of LV tissues from CAD patients and non-CAD controls. (**A**) Heatmap of 173 differentially expressed genes (FDR < 0.1). (**B**) STRING network analysis revealing 86 edges (i.e., interactions) between the 173 differentially expressed genes in CAD patients (n = 8) compared with non-CAD controls (n = 7). Molecular mediators within the GO:0009892 (Negative Regulation of Metabolic Process) are annotated as red circles.

To begin elucidating the molecular networks that were altered in the CAD patients, RNA-sequencing was performed on total RNA extracted from LV tissues of healthy non-CAD control hearts (n = 7) and CAD patients (n = 8). Compared to healthy controls, a total of 173 differentially expressed genes (FDR < 0.1) were detected in the LV of CAD hearts (Fig. 8A and Supp Table 2). As expected, gene network analysis using IPA revealed that the greatest enrichment of gene ontologies (GO) between CAD and non-CAD hearts were related to the cardiovascular system (43 genes). At the molecular network level, STRING analysis indicated that the 173 differentially expressed genes from a cohesive network (86 interactions; p = 0.003), of which the most significantly enriched GO was related to regulation of metabolic processes (GO: 0009892; FDR < 0.05) (Fig. 8B and Supp Table 3). These data support our functional data of a metabolic shift and impaired energy production that we observed in the LV tissue of CAD patients, and provides multiple molecular mediator candidates for future analysis.

## Discussion

To the best of our knowledge, this is the first study to fully characterize changes in mitochondrial respiration and _mt_DNA integrity and its detailed effect on mitochondrial metabolism within the human heart from subjects with and without CAD. There are five major novel findings of this study. First, we show decreased _mt_DNA integrity in subjects with clinical diagnosis of CAD. Second, regulators of mitochondrial networks (fission and fusion) are both upregulated in CAD. Third, we show that decreased levels of ATP and NAD + in the CAD hearts are accompanied by a decreased respiratory capacity of mitochondria isolated from CAD hearts. Fourth, reduced mitochondrial respiratory activity is compensated by an increase in glycolytic flux in the hearts from CAD subjects. Lastly, the mitochondrial genome and the RNAseq analyses confirm a general metabolic defect in the hearts from CAD subjects.

The human heart must continuously pump blood to supply oxygen and nutrients to tissue; as such, it has substantial energy demands, even at rest^42^. Most of this energy is supplied by the cell’s powerhouse, the mitochondria, and mitochondrial energy production is thus essential for normal cardiac function. Thus, it is no surprise that mitochondrial dysfunction is associated with many cardiovascular diseases (reviewed in^43^). Mitochondrial ATP production requires the universal cofactor NAD^+^. Studies have shown that NAD^+^ levels are limiting for mitochondrial energy production. In this study, we show that CAD hearts display a drastic decrease in NAD^+^ and ATP levels. Compared to previous reports, our observed ATP levels were surprisingly low. A possible explanation is we have evaluated ATP levels directly *ex vivo* using frozen heart tissues vs. previous studies, which quantified phosphocreatine to ATP ratios *in vivo*^44,45^. Hence, we can only speculate about the absolute levels of ATP in the CAD hearts as our analysis was purely comparative.

Mitochondrial OXPHOS supplies ATP for most mammalian cells^46^. In the human heart, 90% of cellular ATP is produced by OXPHOS; therefore, a defect in this pathway represents a key concern in terms of cardiac energy production^47–49^. In accordance with the reduced levels of ATP observed in CAD hearts, mitochondria isolated from fresh human hearts of subjects with CAD contained a significant OXPHOS defect, as indicated by a decrease in mitochondrial respiration in response to two major substrates used to activate the OXPHOS pathway. Although, the overall protein expression of OXPHOS subunits were upregulated in the CAD hearts, a decrease in the activities of complexes I, II and III was observed in the mitochondrial lysates from CAD subjects, suggesting a functional deficit in OXPHOS-related proteins despite a compensatory increase in protein expression.

We did not measure the level of ROS in the present study nor its effect on the ETC complexes’ respective activities; however, higher oxidative stress has been widely reported in patients with CAD and atherosclerosis^50–57^. Because of their instability and very short half-life, the means used to measure ROS are limited, especially in clinical conditions. Most of the studies emphasis on detecting the stable downstream products of free radicals in body fluids, such as oxidized DNA, advanced oxidation protein products, lipids, or assess altered defense mechanisms (superoxide dismutase levels, oxidized/reduced glutathione ratio). Previous studies showed that plasma levels of oxidized LDL^51,52^, malondialdehyde^51^, and advanced oxidation protein products^57^ were significantly higher in patients with CAD than in those without CAD. Of relevance to the data of the present study, impairment of complexes I and III due to ROS have been previously reported^58,59^ and suggested to cause electron leak from the ETC, leading to a feed-forward cycle of oxygen radical-induced damage to mitochondrial membrane components. Moreover, oxidative post-translational modification of complex I and complex II has been shown to affect enzymatic catalysis and enzyme-mediated ROS production^60–65^, which in turn contributes to compromised mitochondrial respiration and ATP synthesis, and in the pathogenesis of many diseases, including diabetes and heart failure^2,6,66–69^.

Complex I plays a major role in energy metabolism as the main consumer of NADH in OXPHOS pathway, and defects in complex I have been linked to disorders in highly metabolizing organs like the heart^70^. The used in-gel assays demonstrated decreased complex I activity, yet we observed an increase in complex I subunit expression by immunoblotting and RNAseq data (Supp Table 2) (for example: NUDFA10, NUDFA5, NUDFF7, NUDFB1, etc.) and assembly into respirasomes. Interestingly, we found that the ability of complex I to accept electrons from NADH (NADH oxidase activity) was unchanged in CAD patients, but complex I was more likely to be in its de-active state and less likely to pass electrons to ubiquinone and complex III^71,72^. It is possible that the oxidative stress (increased ROS) induced by CAD can exacerbate this deactivation of complex I in the heart^73,74^. In addition, the decrease in electron transfer from complex I to III may explain decreases in activity of these complexes in in-gel assays and of maximal oxygen consumption experiments. Furthermore, the substantial decrease in the ETC activity may induce a compensatory upregulation of the respiratory complexes and their assembly into respirasomes, but this upregulation is futile when complex I remains inactive.

Beta-oxidation of fatty acids contributes the majority of carbon substrates for ATP production^75^ through OXPHOS, and glycolysis contributes only about 5% of the total ATP generated in cardiac aerobic metabolism^76^. However, the heart possesses tremendous metabolic flexibility and can utilize glucose, lactate and ketones to support its demand for ATP generation. Preference in substrate utilization can change in response to altered metabolic pathways. It is well established that in the ischemic heart, mitochondrial metabolic dysfunction caused by reduced oxygen delivery to the heart results in a decrease in ATP formation by OXPHOS^77^. The reduction in ATP formation through oxygen consumption induces glycolysis, glucose uptake and glycogen breakdown, leading to an increase in the contribution of glycolysis to ATP production^78^. In addition, studies have shown an increased activity of lactate dehydrogenase, the enzyme responsible for the conversion of pyruvate to lactate, as well as the elevated efflux of lactate from the hypertrophied myocardium^79–81^. Consistent with these findings, we observed increased utilization of glycolytic pathways that coincided with altered gene expression in key metabolic pathways in CAD hearts. It is possible that, in CAD hearts, a significant decrease in oxygen supply due to narrowing of major arteries stimulates a cardiac metabolic switch from the aerobic ATP production via OXPHOS to an anaerobic pathway through glycolysis^82^. On the other hand, we cannot exclude the effect of oxidative stress on post-translational modification of ETC complexes and resulting changes in enzymatic activities.

The role of fission and fusion as regulators of normal mitochondrial function have been the subject of debate and controversy for some time. Mitochondrial structure and networks are very dynamic and can rapidly respond to changes in energy demand and extra cellular stress^83,84^. Mitochondrial dynamics are tightly regulated through the balanced processes of fusion and fission and have significant impact on OXPHOS, _mt_DNA stability, mitochondrial quality and ROS generation^85^. Oxidative stress has been shown as one of the stimuli for an increased level of mitochondrial fusion^86–88^. The data of the present study display an increase in the markers of fusion, MFN1, MFN2 and OPA1 in the CAD hearts, indicating a stimulation of the mitochondrial fusion within the CAD hearts. Notably, our data reveal an increase in the markers of fission, p-DRP1 and t-DRP1, which leads us to believe that the mitochondria are in constant changes of morphology to adapt to the level of stress related to CAD. When mitochondrial integrity is compromised, mitochondria fuse to isolate defective _mt_DNA gene products from healthy nearby mitochondria^89^. Due to its proximity and vulnerability to mitochondrial ROS, an accumulation of mutations and damage to _mt_DNA has been reported in subjects with CAD^2,6,68^ and in animal model of myocardial infarction^69^. In addition to these observations, LV samples from CAD subjects used in this study contained a significant increase in _mt_DNA common deletions and _mt_DNA damage. Consistent with this hypothesis, studies have demonstrated that cellular stresses such as myocardial ischemia precipitate increased mitochondrial fission^90–93^. Interestingly, hypoxia has been shown to induce a mitochondrial turnover through processes of mitophagy and mitochondrial biogenesis^94^. It is possible that the cardiac mitochondria used in this study exhibit a similar adaptive response.

### Study limitations

Due to common restrictions in working with human specimens, several limitations are worth noting in the current study. First, due to limited access to human cardiac tissue, we cannot control underlying and contributing clinical risk factors. As a result, we cannot conclude whether mitochondrial defects promote development of CAD or if CAD leads to mitochondrial defects in the human heart. Second, since the objective of the study is to assess the metabolic profile of diseased subjects (CAD) in comparison to “healthy” subjects, the availability of cardiac specimens from such individuals at relatively older age is very limited. Although we previously reported that vascular function phenotype associated with CAD is independent of aging^16^ we cannot exclude age as a confounding factor of our current observations. A similar situation presents itself with other present risk factors such as hypertension or diabetes. Along these lines, studies involving human specimens suffer from heterogeneity among subjects. Tissues were collected from subjects with diverse genetic backgrounds and lacked full clinical information and medications that might interfere with gene expression or mitochondrial proteins. The sample size of our current study does not allow for statistical evaluation of contributing factors such as race, or ethnic background. Age, one of the key risk factors for development of CAD, was significantly higher in the CAD subjects compared to the Non-CAD (CAD: 59 ± 8*, n = 22; Non-CAD: 49 ± 14 n = 35; *p < 0.05 in CAD vs Non-CAD subjects). In addition, the gender disparity is higher in Non-CAD samples with fewer men represented than women (Non-CAD: Men 32% and women 68%; CAD: Men 46% and women 54%).

We are also aware that mitochondrial structure and dynamics should not be solely assessed by protein expression, as this does not offer a direct measure and thus we can only speculate on the fusion/fission state of mitochondria in human heart tissues. Current technologies do not allow for a reliable means to analyze mitochondrial networks in whole tissue. The use of Electron Microscopy is considered the gold standard to evaluate mitochondrial networks, but due to the two-dimensional nature of Electron Microscopy, one cannot accurately quantify mitochondrial networks in general as recantly demonstrated by us [please insert PMID:30476208], which should also be aplicable for *ex vivo* human tissues. The same problem presents itself in assessing glycolysis. It is not feasible to measure CO_2_ formation from labelled glucose injected directly *in vivo* in order to draw conclusion about the glycolytic state in CAD. Future direct mechanistic studies such as rescue experiments include use of animal models necessary to definitely confirm this connection.

## Conclusions

Our main findings describe an overall decline in mitochondrial function and energy production in patients with CAD. Our data show an elevation in _mt_DNA deletions and damage accompanied by a decrease in OXPHOS and ETC complex activities; although we do not provide direct evidence of causality, this relationship has been previously suggested^2,6^. Furthermore, our data suggest decreased mitochondrial respiration is at least in part a result of decreased activation of mitochondrial supercomplexes in the ETC. The decline in mitochondrial OXPHOS activity and ATP production contributes to and promotes a metabolic shift towards glycolysis which has previously been reported in subjects with chronic heart failure^14^. Additional work is required to elucidate the contribution of mitochondrial phenotype in cardiac disease and whether _mt_DNA-targeted therapies could be considered for preventing cardiovascular diseases.

## Supplementary information

Supplemental material
