# Supplementary material for: Mitochondrial Oxidative Phosphorylation defect in the Heart of Subjects with Coronary Artery Disease
Source: Sci Rep. 2019 May 20;9:7623. doi: 10.1038/s41598-019-43761-y (PMC6527853; doi:10.1038/s41598-019-43761-y)

Mitochondrial Oxidative Phosphorylation defect in the Heart of Subjects with Coronary Artery Disease

Karima Ait-Aissa*, Scott C. Blaszak, Gisela Beutner, Shirng-Wern Tsaih, Garrett Morgan, Janine H. Santos, Michael J. Flister, David L. Joyce, Amadou K.S. Camara, David D. Gutterman, Anthony J. Donato, George A. Porter, Jr., Andreas M. Beyer*

Supplemental information

Supp Table 1: Patient demographics for heart tissues. *$p<0.05$ CAD patients vs Non-CAD patients.

Supp Table 2: Total differentially expressed genes detected in the LV of CAD (n=8) in comparison to Non-CAD (n = 7), total of 173 genes, FDR < 0.1.

Supp Table 3: Total differentially expressed genes related to regulation of metabolic processes. String analysis, 86 interactions, $p = 0.003$, GO: 0009892; FDR < 0.05.

Supp Table 4: In-gel assay ETC complexes activity reagents.

Supp Figure 1: Supplemental information to Figure 5

Supp Figure 2: ATP and NAD+ levels measured in right and left atria and right ventricles of human hearts; n=15 in each group; *$p<0.05$ vs Non-CAD hearts.

Supp Figure 3: Full immuno-blot against OXPHOS antibody cocktail in LV lysates from CAD and Non-CAD subjects.

Supp Table 1: Patient demographics for heart tissues

Subject demographics	Non-CAD (22)	CAD (35)
Characteristics		
Sex, Male/Female	7 / 15	16 / 19
Age, years (mean+/- SD	49 ± 14	59 ± 8
Body Mass Index	29± 7	33± 7
Underlying conditions		
Coronary Artery Disease	0	35
Hypertension	4	22
Diabetes Mellitus	1	13
Hypercholesterolemia	0	4
Myocardial Infarction	2	7
None of the above	15	0

Supplemental Table 2. Differentially expressed genes in CAD patients compared with healthy controls

Gene Name	Chr	Start	End	log2 Fold Change	FDR
LINC01128	1	825,138	859,446	0.59	0.099
TNFRSF4	1	1,211,326	1,214,138	-0.86	0.040
EPHB2	1	22,710,839	22,921,500	-0.91	0.057
RNF19B	1	32,936,445	32,964,685	-0.51	0.076
SLC6A9	1	43,991,500	44,031,467	-0.78	0.093
CYP4B1	1	46,757,838	46,819,413	1.09	0.008
CYP4Z1	1	47,067,488	47,118,319	1.04	0.021
TAL1	1	47,216,290	47,232,220	0.74	0.044
DHCR24	1	54,849,633	54,887,218	-0.90	0.057
FAM69A	1	92,832,737	92,961,522	-0.56	0.063
RGS4	1	163,068,775	163,076,802	-0.87	0.071
RP4-798P15.3	1	177,928,788	178,038,007	0.84	0.076
LAD1	1	201,373,244	201,399,915	-0.73	0.076
PRELP	1	203,475,828	203,491,352	0.87	0.057
ATP2B4	1	203,626,561	203,744,081	-0.68	0.063
SERTAD4	1	210,232,799	210,246,631	-0.86	0.063
TGFB2-OT1	1	218,442,626	218,443,996	-0.84	0.073
ACTA1	1	229,431,245	229,434,098	-0.92	0.057
TPO	2	1,374,223	1,543,711	0.78	0.034
POMC	2	25,160,853	25,168,903	0.19	0.060
MSH6	2	47,695,530	47,810,101	-0.39	0.093
UGP2	2	63,840,940	63,891,562	-0.71	0.055
SMYD5	2	73,214,222	73,227,237	-0.58	0.062
IL18R1	2	102,311,529	102,398,775	0.80	0.098
MYO7B	2	127,535,802	127,637,729	0.91	0.048
RP11-286H15.1	2	127,625,997	127,626,848	0.87	0.062
DPP4	2	161,992,241	162,074,542	0.67	0.071
NOSTRIN	2	168,786,539	168,865,514	0.73	0.035
AOX1	2	200,585,868	200,677,064	1.23	0.001
TUBA4A	2	219,249,711	219,278,170	-0.89	0.055
PTPRN	2	219,289,623	219,309,648	-0.52	0.055
SERPINE2	2	223,975,112	224,039,319	-0.89	0.060
ACKR3	2	236,567,787	236,582,358	0.63	0.076
LMCD1	3	8,501,707	8,574,673	-0.86	0.062
GALNT15	3	16,174,649	16,231,992	0.98	0.021
EXOG	3	38,496,127	38,542,161	0.53	0.097
KLHL40	3	42,685,519	42,692,544	-0.88	0.044
IP6K2	3	48,688,003	48,740,353	0.56	0.041
FAM212A	3	49,803,254	49,805,030	-0.76	0.063
DNASE1L3	3	58,192,257	58,214,697	0.82	0.093
FAM107A	3	58,564,117	58,627,610	1.00	0.001
NFKBIZ	3	101,827,991	101,861,022	0.65	0.057
KLF15	3	126,342,635	126,357,442	0.75	0.037

Gene Name	Chr	start	end	log2 Fold Change	FDR
RP11-451G4.2	3	155,290,229	155,293,775	0.77	0.063
LINC00886	3	156,747,346	156,817,062	0.89	0.041
PTX3	3	157,436,789	157,443,628	0.47	0.066
ETV5	3	186,046,308	186,110,318	-0.76	0.097
CXCL10	4	76,021,117	76,023,497	-0.94	0.001
LINC01088	4	78,971,748	79,308,798	1.13	0.007
RP11-766F14.2	4	99,636,529	99,654,648	-0.65	0.007
RP11-420A23.1	4	128,292,751	128,519,394	0.84	0.071
TMEM154	4	152,618,632	152,680,165	0.72	0.097
SFRP2	4	153,780,592	153,789,120	1.28	0.001
TLL1	4	165,873,258	166,103,895	-0.90	0.057
DAP	5	10,679,230	10,761,272	-0.61	0.037
LIFR	5	38,474,963	38,608,354	0.65	0.093
C5orf64	5	61,637,708	61,751,763	0.91	0.047
PPWD1	5	65,563,236	65,587,549	0.39	0.084
XRCC4	5	83,077,498	83,353,787	-0.90	0.057
MARCH3	5	126,867,714	127,030,808	-0.77	0.057
MYOT	5	137,867,791	137,887,851	-0.80	0.091
CNOT8	5	154,857,553	154,876,793	0.34	0.072
NEDD9	6	11,183,298	11,382,348	0.75	0.037
SOX4	6	21,592,768	21,598,619	-0.74	0.066
HIST1H2BD	6	26,158,146	26,171,349	0.74	0.071
TUBB	6	30,720,201	30,725,426	-0.77	0.063
IER3	6	30,743,199	30,744,554	-0.95	0.014
CNR1	6	88,139,864	88,166,359	0.82	0.093
TUBE1	6	112,070,777	112,087,529	0.70	0.016
ZDHHC14	6	157,381,133	157,678,146	-0.51	0.057
LFNG	7	2,512,529	2,529,177	-0.75	0.040
TRIL	7	28,953,358	28,958,292	-1.22	0.001
HSPB1	7	76,302,544	76,304,295	-0.85	0.057
LRRC17	7	102,912,991	102,944,949	-1.02	0.024
FLNC	7	128,830,377	128,859,274	-0.81	0.076
KIAA1549	7	138,831,381	138,981,318	0.84	0.016
LOXL2	8	23,297,189	23,425,328	-0.74	0.099
STC1	8	23,841,915	23,854,807	0.84	0.075
DPYSL2	8	26,514,022	26,658,178	0.41	0.093
TACC1	8	38,728,186	38,853,028	0.58	0.048
PDE7A	8	65,717,510	65,842,322	0.49	0.057
MSC	8	71,841,549	71,844,468	-0.80	0.097
DENND3	8	141,117,278	141,195,808	0.73	0.001
TESK1	9	35,605,305	35,610,041	-0.55	0.093
MAMDC2	9	70,043,581	70,226,970	0.75	0.093
KLF9	9	70,384,597	70,414,624	0.62	0.098
FIBCD1	9	130,902,438	130,939,286	-0.76	0.057
CLIC3	9	136,994,635	136,996,803	0.82	0.095

Gene Name	Chr	start	end	log2 Fold Change	FDR
FZD8	10	35,638,249	35,642,278	-0.77	0.057
ZNF37BP	10	42,513,510	42,552,822	0.59	0.057
RP11-96C23.11	10	87,001,636	87,009,905	0.70	0.099
LOXL4	10	98,247,690	98,268,250	-0.77	0.076
APLNR	11	57,233,577	57,237,314	-1.01	0.012
TIGD3	11	65,354,767	65,357,613	0.84	0.093
PACS1	11	66,070,363	66,244,747	-0.61	0.076
CDK2AP2	11	67,506,497	67,508,649	-0.84	0.057
P2RY6	11	73,264,505	73,298,617	-0.81	0.097
RP11-21L23.2	11	76,800,364	76,804,555	0.99	0.035
BIRC3	11	102,317,450	102,339,403	0.73	0.080
MMP8	11	102,711,795	102,727,050	0.05	0.063
UBASH3B	11	122,655,675	122,814,473	-0.83	0.095
COPS7A	12	6,723,741	6,731,875	-0.42	0.057
CLEC4E	12	8,533,305	8,540,963	0.77	0.095
BHLHE41	12	26,120,026	26,125,127	-0.80	0.031
RP11-161H23.5	12	49,265,156	49,273,306	-0.80	0.057
GRASP	12	52,006,940	52,015,889	0.83	0.040
TNS2	12	53,046,969	53,064,372	0.57	0.035
TMEM198B	12	55,829,608	55,836,246	0.74	0.035
RBMS2	12	56,521,929	56,596,196	0.42	0.072
IRAK3	12	66,188,879	66,254,622	0.63	0.093
APPL2	12	105,173,296	105,236,238	0.49	0.057
TMEM119	12	108,589,846	108,598,320	-0.87	0.041
ANKRD13A	12	109,999,186	110,039,763	-0.82	0.066
WSB2	12	118,032,694	118,062,430	-0.63	0.017
CABP1	12	120,640,552	120,667,324	0.80	0.040
SLC7A1	13	29,509,410	29,595,688	-0.98	0.034
DACT1	14	58,633,967	58,648,321	-1.18	0.001
SLC24A4	14	92,322,581	92,501,483	0.90	0.057
C15orf41	15	36,579,611	36,810,248	-0.70	0.076
RP11-1069G10.2	15	62,895,812	62,899,543	-0.88	0.071
CD276	15	73,683,966	73,714,518	-0.68	0.076
LOXL1-AS1	15	73,908,071	73,928,248	-0.90	0.055
LOXL1	15	73,925,989	73,952,137	-0.83	0.095
LMAN1L	15	74,812,716	74,825,758	1.21	0.001
EFL1	15	82,130,230	82,262,763	-0.50	0.097
TNFRSF12A	16	3,018,445	3,022,383	-0.97	0.035
C16orf89	16	5,044,122	5,066,110	0.87	0.060
MT1JP	16	56,635,739	56,637,086	1.08	0.014
MT1X	16	56,682,424	56,684,196	0.84	0.057
PLLP	16	57,248,547	57,284,687	0.66	0.040
SLC7A6	16	68,264,516	68,301,823	0.56	0.036
KLHL36	16	84,648,525	84,667,686	0.60	0.071
MAP2K3	17	21,284,672	21,315,240	-0.77	0.093

Gene Name	Chr	start	end	log2 Fold Change	FDR
CCL11	17	34,285,668	34,288,334	-0.86	0.064
PNMT	17	39,667,981	39,670,475	0.85	0.057
RP11-242D8.1	17	43,148,368	43,171,037	0.58	0.057
ITGA3	17	50,055,968	50,090,481	-0.87	0.043
COL1A1	17	50,183,289	50,201,632	-0.90	0.057
ABCC3	17	50,634,777	50,692,252	-0.83	0.077
ACE	17	63,477,061	63,498,380	-1.30	3.1E-06
BAIAP2	17	81,035,122	81,117,432	0.75	0.037
MRO	18	50,795,120	50,825,402	0.82	0.097
GADD45B	19	2,476,122	2,478,259	0.70	0.088
FZR1	19	3,506,273	3,538,330	-0.35	0.063
PLIN3	19	4,838,341	4,867,768	-0.76	0.048
PPAN-P2RY11	19	10,106,223	10,114,780	0.45	0.057
RGL3	19	11,384,341	11,419,342	0.75	0.072
NOTCH3	19	15,159,038	15,200,981	-0.69	0.099
CPAMD8	19	16,892,947	17,026,815	0.93	0.001
ARRDC2	19	18,001,132	18,014,102	0.76	0.047
ZNF676	19	22,179,091	22,196,951	0.80	0.071
RELB	19	45,001,430	45,038,198	-0.85	0.048
DMPK	19	45,769,717	45,782,552	-0.69	0.093
HIF3A	19	46,297,046	46,343,433	0.76	0.002
IZUMO1	19	48,740,852	48,746,909	0.84	0.093
PTPRH	19	55,181,248	55,209,506	-1.06	0.017
REM1	20	31,475,293	31,484,905	-0.84	0.034
FOXS1	20	31,844,301	31,845,619	-0.87	0.057
SIK1	21	43,414,515	43,427,128	0.38	0.040
PRMT2	21	46,635,167	46,665,124	-0.45	0.084
RASL10A	22	29,312,933	29,319,679	0.82	0.097
SLC5A1	22	32,043,032	32,113,029	0.79	0.063
TIMP3	22	32,801,701	32,863,043	0.69	0.057
TRIOBP	22	37,697,004	37,776,556	-0.35	0.087
APOBEC3A	22	38,952,741	38,992,778	0.85	0.076
ZRSR2	X	15,790,472	15,823,260	0.37	0.097
CDKL5	X	18,425,583	18,653,629	0.60	0.057
MAGED4B	X	52,061,827	52,069,248	-0.83	0.097
ARMCX6	X	101,615,118	101,618,001	-0.62	0.016
TSC22D3	X	107,713,221	107,777,342	1.00	0.001
MBNL3	X	132,369,317	132,489,968	0.85	0.042
FHL1	X	136,146,702	136,211,359	-1.01	0.021
AC004556.1	KI270721.1	2585	11802	0.05	4.23E-08

Supplemental Table 3. Differentially expressed genes that are linked with metabolic

Gene Name	Chr	Start	End	log2 Fold Change	FDR
TNFRSF4	1	1211326	1214138	-0.864	0.040
TAL1	1	47216290	47232220	0.739	0.044
DHCR24	1	54849633	54887218	-0.899	0.057
RGS4	1	163068775	163076802	-0.867	0.071
ATP2B4	1	203626561	203744081	-0.681	0.063
MSH6	2	47695530	47810101	-0.387	0.093
NOSTRIN	2	168786539	168865514	0.735	0.035
SERPINE2	2	223975112	224039319	-0.888	0.060
KLHL40	3	42685519	42692544	-0.881	0.044
KLF15	3	126342635	126357442	0.746	0.037
PTX3	3	157436789	157443628	0.471	0.066
DAP	5	10679230	10761272	-0.606	0.037
CNOT8	5	154857553	154876793	0.345	0.072
SOX4	6	21592768	21598619	-0.737	0.066
IER3	6	30743199	30744554	-0.954	0.014
CNR1	6	88139864	88166359	0.818	0.093
HSPB1	7	76302544	76304295	-0.847	0.057
LOXL2	8	23297189	23425328	-0.742	0.099
FZD8	10	35638249	35642278	-0.773	0.057
BIRC3	11	102317450	102339403	0.730	0.080
UBASH3B	11	122655675	122814473	-0.830	0.095
BHLHE41	12	26120026	26125127	-0.797	0.031
IRAK3	12	66188879	66254622	0.632	0.093
CABP1	12	120640552	120667324	0.800	0.040
DACT1	14	58633967	58648321	-1.182	0.001
CD276	15	73683966	73714518	-0.682	0.076
GADD45B	19	2476122	2478259	0.702	0.088
FZR1	19	3506273	3538330	-0.346	0.063
CPAMD8	19	16892947	17026815	0.926	0.001
RELB	19	45001430	45038198	-0.846	0.048
HIF3A	19	46297046	46343433	0.757	0.002
FOXS1	20	31844301	31845619	-0.872	0.057
SIK1	21	43414515	43427128	0.384	0.040
PRMT2	21	46635167	46665124	-0.454	0.084
TIMP3	22	32801701	32863043	0.695	0.057
TSC22D3	X	107713221	107777342	0.995	0.001

Supp Table 4: In-gel assay ETC complexes activity reagents.

Complex	Substrate
**Complex I**	NADH (10mg/mL), nitrotetrazolium blue (2.5mg)
**Complex II**	Sodium succinate (1M), Phenazinmethosulfate (250mM), nitrotetrazolium blue (2.5mg)
**Complex III**	Diaminobenzidine (0.5mg/mL), Sodium Phosphate buffer (50mM)
**Complex IV**	Horse heart Cytochrome C (5mM), diaminobenzidine (0.5mg/mL)
**Complex V**	ATP (8mM)

Supplemental Figure 1

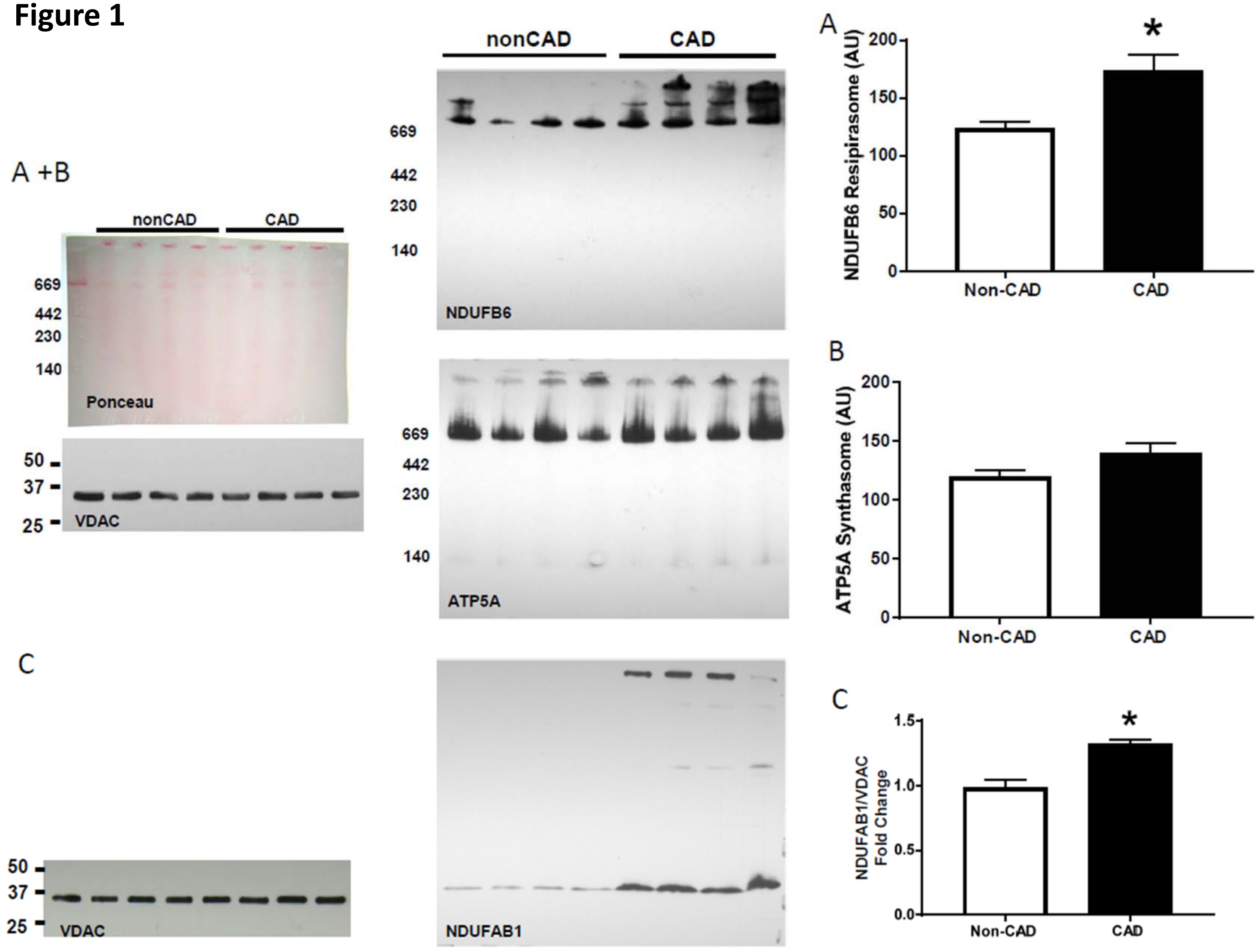

Supplemental Figure 2

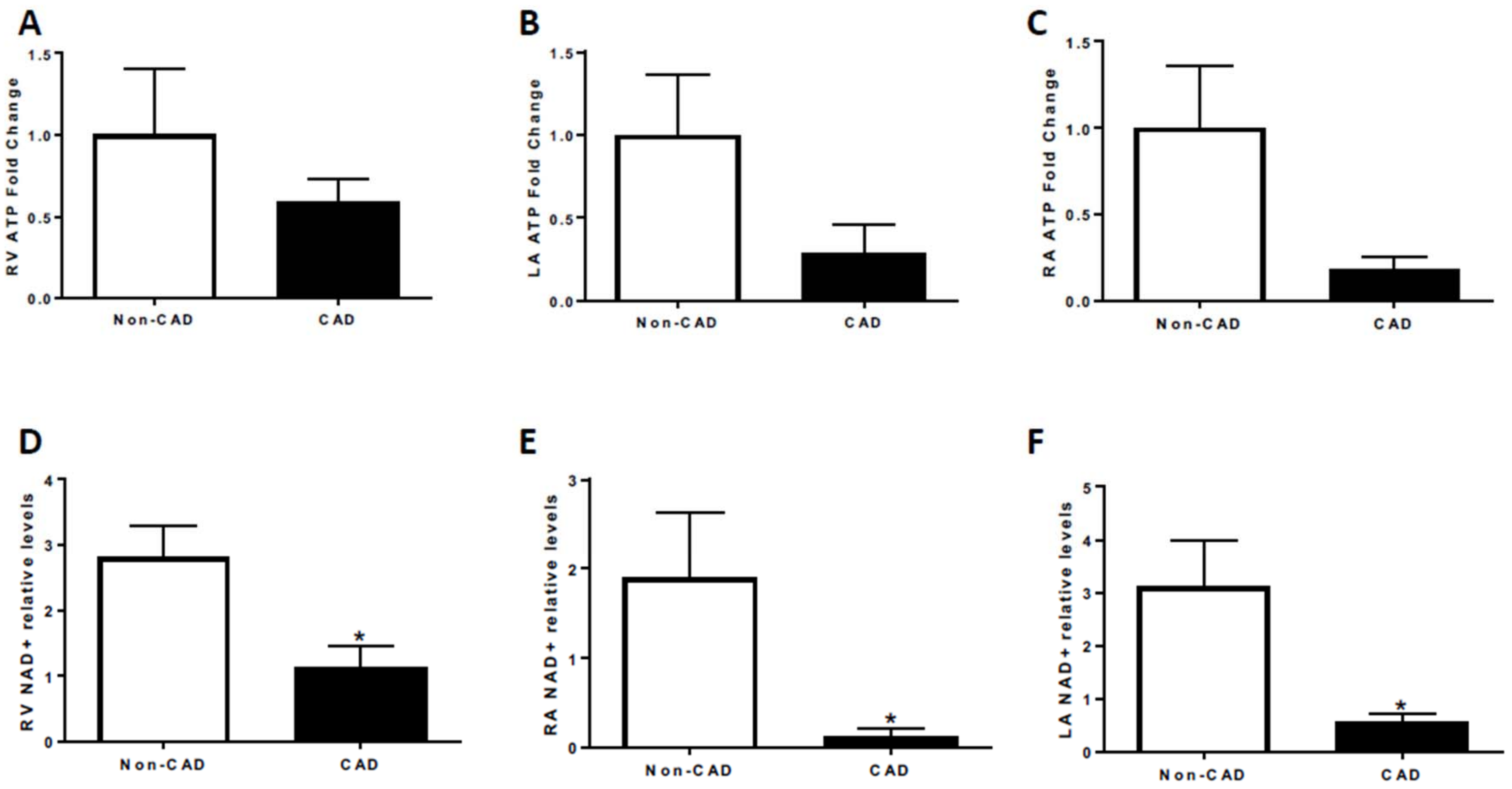

Supplemental Figure 3

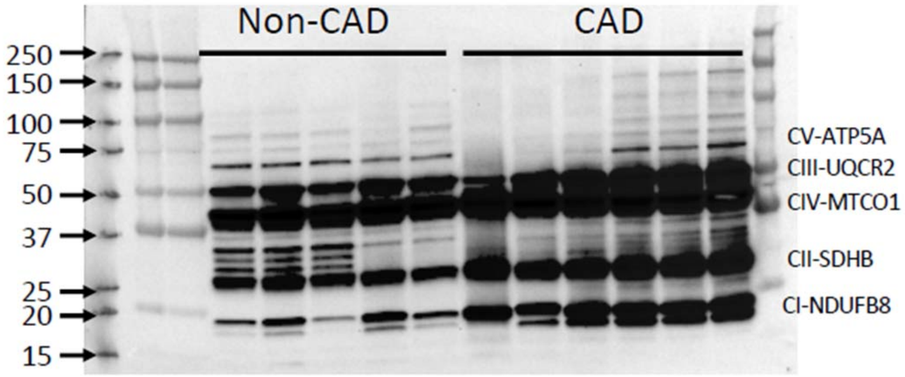

Supplement: Supplementary file 1 — Supplemental material [file 41598_2019_43761_MOESM1_ESM.pdf]
